# Alterations in Muscle Force Control With Aging: Is There a Modulatory Effect of Lifelong Physical Activity?

**DOI:** 10.3389/fspor.2022.817770

**Published:** 2022-03-22

**Authors:** Jamie Pethick, Mathew Piasecki

**Affiliations:** ^1^School of Sport, Rehabilitation and Exercise Sciences, University of Essex, Colchester, United Kingdom; ^2^Centre of Metabolism, Ageing and Physiology (COMAP), MRC-Versus Arthritis Centre for Musculoskeletal Ageing Research, National Institute for Health Research (NIHR) Nottingham Biomedical Research Centre, School of Medicine, University of Nottingham, Nottingham, United Kingdom

**Keywords:** aging, force control, muscle, motor unit (MU), Masters athletes, physical activity

## Abstract

Recent technological developments have enabled significant advances in our understanding of the ability to voluntarily control muscle force output. The fluctuations inherent to muscle force output can be quantified according to both their magnitude and temporal structure (or “complexity”), with such quantification facilitating comparison of force control between distinct populations. In comparison to young adults, older adults exhibit an increase in the magnitude (i.e., decreased steadiness) and a decrease in the complexity (i.e., decreased adaptability) of force fluctuations, both of which are indicative of a loss of force control. There remain, however, key gaps in knowledge that limit our interpretation of this age-related loss of force control. One such gap relates to the effect of lifelong physical activity on force control. To date, research on aging and force control has largely been conducted on inactive or moderately active older adults. However, high levels of lifelong physical activity, such as that exhibited by Masters athletes, have been shown to have protective effects on the function and morphology of the neuromuscular system. Some of these effects (e.g., on impaired inhibitory transmission in the motor cortex and on motor unit discharge rates) have the potential to attenuate the age-related loss of force control, while others (e.g., greater motor unit remodeling capacity) have the potential to worsen it. We therefore propose that, in order to progress our knowledge of the effects of aging on force control, future studies must consider the potential modulatory effect of lifelong physical activity.

## Introduction

Voluntary control of skeletal muscle is accomplished by precise activation of motor unit (MU) populations mediated by excitatory and inhibitory processes (Enoka and Farina, 2021). When an individual attempts to maintain a constant force during an isometric or (near) isotonic contraction, muscle force is not constant; rather, it fluctuates around an average value (Enoka et al., 2003). Such fluctuations in muscle force were long regarded as unwanted noise but are now recognized as a source of information about the neural mechanisms underlying force control (Enoka and Farina, 2021). Moreover, metrics that quantify various aspects of muscle force fluctuations (classically their magnitude and more recently their temporal structure or “complexity”) can be used as a paradigm to compare force control between populations.

One comparison that has received much attention is that between young and old adults. Numerous studies have demonstrated that aging from adulthood to senescence decreases the ability to generate task-relevant and precise levels of force, i.e., results in a loss of force control (Galganski et al., 1993; Vaillancourt and Newell, 2003). The mechanistic basis of the age-related loss of force control is multifactorial and has been speculated to relate to alterations in the neural input to MUs and consequent changes in their discharge properties (Castronovo et al., 2018) and to a loss of MUs and consequent MU remodeling (Challis, 2006). Recent evidence has suggested that this age-related loss of force control contributes to a reduced ability to perform activities of daily living (ADLs; Feeney et al., 2018; Davis et al., 2020).

The effects of aging on muscle force control, as well as the neuromuscular structures and processes underlying it, are, however, not uniform amongst older adults (Degens and Korhonen, 2012) and inter-subject variability is greater than in young adults (Vanden Noven et al., 2014). This non-uniformity is partly genetic but more generally reflects external lifestyle factors, in particular physical activity (Lazarus and Harridge, 2018). Indeed, physical inactivity is the most important factor accelerating age-related declines in physiological function, while physical activity is considered the most important factor in slowing such decline (Booth et al., 2011). It is evident that a wide continuum of activity levels exists in older adults; ranging from those who are very sedentary (evidenced by prolonged sitting) to those who maintain high levels of training and are still competitive (i.e., Masters) athletes (Lazarus and Harridge, 2017).

Masters athletes have been proposed to represent the ideal biological model to study the effects of inherent (or “healthy”) aging, as they are unaffected by the confounding effects of inactivity (Lazarus and Harridge, 2017). Studies involving Masters athletes have demonstrated that lifelong physical activity can minimize age-related losses of functional capacity, e.g., strength (McKendry et al., 2018) and balance (Leightley et al., 2017), in comparison to age-matched controls. Although data on the preservation of MU number in Masters athletes is uncertain, showing evidence for (Power et al., 2010) and against (Piasecki et al., 2016, 2019), numerous data suggests greater reinnervation capacity of denervated muscle fibers in Masters athletes compared to age-matched controls (Piasecki et al., 2019; Sonjak et al., 2019). To date, however, research on aging and force control has focused on heterogeneous groups of sedentary to moderately active older adults and has often excluded more active adults (Oomen and van Dieën, 2017). It is, therefore, reasonable to speculate that our current understanding of age-related changes in force control represents a combination of the inherent aging process interacting with the pathophysiological consequences of inactivity (Harridge and Lazarus, 2017).

The aim of this perspective is to provide a brief overview of the current state of the art, and recent developments, regarding how muscle force control is affected by aging and to present an argument that, to progress this field, future research *must* compare individuals of differing physical activity status in order to disentangle the inherent effects of aging on force control from the combined effects of inherent aging and inactivity.

## State of the Art and Recent Developments

Muscle force control is typically quantified using targeted submaximal isometric contractions (at a percentage of participants' maximal voluntary contraction, MVC), during which the exerted force fluctuates around the imposed target. Classically, these fluctuations have been quantified according to their magnitude, either in absolute terms using the standard deviation (SD) or in relative terms (i.e., normalized to the mean) using the coefficient of variation (CV; Enoka et al., 2003). The CV of muscle force facilitates comparison between populations differing in strength (e.g., young vs. old adults) and provides a measure of force steadiness (Galganski et al., 1993), with greater values interpreted as decreased steadiness.

Evidence accumulated over the last 30 years has demonstrated that old adults (aged >65 years) exhibit a greater magnitude of force fluctuations (i.e., are less steady) than young adults (aged ~20–30 years; Galganski et al., 1993; Laidlaw et al., 2000). Indeed, a recent meta-analysis found a large pooled effect size (*r* = 0.67) of age on muscle force CV (Oomen and van Dieën, 2017). This age-related decreased ability to control force is, however, dependent on several factors; notably, the contraction intensity and muscle group tested. The decreased ability of older adults to control force is most prominent at low contraction intensities (≤ 10% MVC; Ranganathan et al., 2001; Tracy and Enoka, 2002), which is pertinent as most ADLs performed by older adults typically require only a small fraction of maximal muscle capacity (Tikkanen et al., 2016). Decreases in force control are evident up to ~35% MVC (Oomen and van Dieën, 2017), with smaller or inconsistent effects observed thereafter (Tracy and Enoka, 2002; Tracy, 2007). With regards to muscle group, the most consistent age-related effects have been observed in the index finger abductors (Galganski et al., 1993; Laidlaw et al., 2000). Large pooled effect sizes have also been found for the knee extensors and ankle dorsiflexors (Oomen and van Dieën, 2017), though the elbow flexors seem to be unaffected (Graves et al., 2000).

Over the course of the last decade, research has demonstrated that muscle force fluctuations are strongly correlated with the common component of the cumulative MU spike train (Negro et al., 2009; Thompson et al., 2018). Common synaptic input to motor neurons has, therefore, been postulated to represent the effective neural drive to muscle (Negro and Farina, 2011) and to be the main determinant of force fluctuations (Farina and Negro, 2015). Two recent studies have demonstrated associations between the age-related increase in the magnitude of force fluctuations and variance in common synaptic input. Firstly, Castronovo et al. (2018) observed progressive increases in muscle force CV and variance of common synaptic input to motor neurons across adults with a continuous age distribution from 24 to 75 years. Importantly, the increases in muscle force CV and common synaptic input were significantly correlated. Secondly, Feeney et al. (2018) found that muscle force CV was more strongly associated with variance in common synaptic input to motor neurons for old than young adults. A further mechanism accounting for muscle force fluctuations is the number, and contractile properties, of MUs (Enoka and Farina, 2021). Loss of spinal motor neurons leads to a decline in MU number but an increase in the innervation number of surviving MUs *via* compensatory reinnervation (Piasecki et al., 2016). Consequently, old adults recruit fewer but larger MUs at lower relative forces and firing rates than young adults, leading to greater fluctuations in force output (Spiegel et al., 1996), particularly at low contraction intensities where each MU has a larger contribution to net force (Fuglevand et al., 1993).

A further recent development has been the identification of muscle force CV as a statistically significant explanatory variable for the variance in performance of many ADLs (Enoka and Farina, 2021). The greater magnitude of force fluctuations exhibited by older adults during low intensity contractions has been found to be correlated with poorer performance in tests of manual dexterity (Marmon et al., 2011; Feeney et al., 2018), mobility (Mani et al., 2018), standing balance (Kouzaki and Shinohara, 2010), reactive driving (a task involving responding to unexpected brake lights with accurate and consistent movement; Lodha et al., 2016), and with a greater risk of falls (Carville et al., 2007). Moreover, in many cases greater muscle force CV can explain more of the variance in functional performance than decreased muscle strength (Lodha et al., 2016; Hirono et al., 2021).

Advances in analytical techniques have led to the recognition that muscle force fluctuations can not only be quantified according to their magnitude but also according to their temporal structure or “complexity” (Slifkin and Newell, 1999). Complexity measures quantify the degree of regularity/randomness in an output, e.g., approximate entropy (ApEn; Pincus, 1991), and can identify long-range fractal correlations present in an output e.g., detrended fluctuation analysis (DFA) α (Peng et al., 1994); properties which magnitude-based measures cannot quantify (Goldberger et al., 2002). Using such complexity-based measures, it has been demonstrated that muscle force output is characterized by a statistically irregular temporal structure (Slifkin and Newell, 1999). While magnitude-based measures reflect force steadiness, complexity-based measures are thought to reflect force adaptability; that is, the ability to modulate force output rapidly and accurately in response to task demands (Pethick et al., 2016). Magnitude- and complexity-based measures therefore quantify different aspects of force output and have different functional significance. The ApEn of muscle force output has been suggested to be affected by several confounding variables relating to the contractile properties of motor units, though these effects could be limited by analyzing the ApEn of motor unit discharge rates instead of force output (Dideriksen et al., 2021). Nevertheless, it has been recommended that both magnitude- and complexity-based measures should be used when characterizing force control (Goldberger et al., 2002; Pethick et al., 2021). The first empirical evidence for measures of force complexity being reflective of adaptability has recently been provided by Mear et al. (2022), who demonstrated that muscle force ApEn is a significant explanatory variable for dynamic balance performance, thus providing a parallel with previous research on magnitude-based measures and performance of ADLs.

Since the turn of the millennium, it has been increasingly demonstrated that old adults exhibit less complex force fluctuations than young adults (Vaillancourt and Newell, 2003; Challis, 2006; Fiogbé et al., 2021). This loss of complexity is manifest as a force output in which the fluctuations have become more regular and predictable, and is interpreted as a decrease in the adaptability of force output. It was first demonstrated by Vaillancourt and Newell (2003), who observed a progressive decline in the complexity of index finger abduction force (measured using ApEn and DFA α) during low-intensity isometric contractions from young adults (20–24 years) to old adults (60–69 years) to older-old adults (75–90 years). Recent work has extended this age-related loss of complexity to frailty, with a progressive decrease in ApEn observed when comparing non-frail, pre-frail and frail adults aged over 65 (Carnavale et al., 2020).

## Limitations of Previous Work

A notable gap in the literature to date on muscle force control and aging is a failure to account for, or recognize the effect of, older adult's physical activity status. The majority of studies investigating age-related changes in force control have reported the physical activity of participants as sedentary to moderately active (Oomen and van Dieën, 2017), with some studies excluding more physically active or trained older adults (Graves et al., 2000; Tracy and Enoka, 2002). Only one study has investigated force control in highly active adults (Masters athletes; Piasecki et al., 2021), finding the age-related decrease in force control was still evident. However, the purpose of this study was to investigate the influence of sex, rather than physical activity status, and, as such, the study lacked an age-matched non-athletic control group. Similarly, studies on physical activity and aging have failed to recognize the potential effect on muscle force control. Several studies comparing physically active and inactive older adults have utilized submaximal isometric contractions to measure various MU properties but have not calculated any force control measures that could be derived from such contractions (Power et al., 2016; Piasecki et al., 2019; Jones et al., 2021). To our knowledge, no study has compared the effects of differing physical activity levels amongst older adults on the ability to control force.

Given the maintenance of other aspects of muscle function (and MU properties) in physically active old adults (McKendry et al., 2018; Piasecki et al., 2019), the lack of research on lifelong physical activity and force control represents a significant knowledge gap that limits our understanding of the inherent effects of aging on force control. Moreover, it has been suggested that identification of explanatory variables other than chronological age is necessary to determine the onset and time course of changes in force control across the lifespan (Enoka and Farina, 2021).

## Lifelong Physical Activity as a Modulator of Muscle Force Control?

### Central Adaptations

The synaptic input received by motor neurons arises from afferent feedback, descending cortical and reticulospinal pathways, and neuromodulatory pathways from the brain stem (Enoka and Farina, 2021). Age-related deficits of motor performance are partly explicable by impaired inhibitory processes in pre- and post-synaptic motor cortex regions (Opie et al., 2015). Briefly, central neuronal excitation is governed by excitatory and inhibitory processes, which are largely mediated by glutamate (excitatory) and gamma-aminobutyric acid (GABA; inhibitory) signaling. Although acute excitatory-inhibitory imbalances are known to facilitate neuronal adaptation, it is probable there is a physiological limit on the increase/reduction of each. Studies of aged rodents have reported GABAergic adaptations primarily demonstrating decreased inhibitory activity (Rozycka and Liguz-Lecznar, 2017), and this has been supported in human studies directly associating age-related declines in motor function (finger tapping and reaction time tasks) with impaired GABAergic neurotransmission (Heise et al., 2013). It has been suggested that changes in GABAergic inhibitory activity contribute to modulation of corticospinal excitability for force control (Matsugi, 2019), with the reduced capacity for inhibitory modulation observed with aging being associated with increased neural noise (Manini et al., 2013).

Physical exercise initiates a marked increase in brain oxidative and non-oxidative carbohydrate consumption (Rasmussen et al., 2011), and it is theorized that the non-oxidized carbohydrate is utilized for *de novo* synthesis of neurotransmitters, including glutamate and GABA which can be synthesized from carbohydrate substrates and amino acids. This has been supported with the use of proton magnetic resonance spectroscopy to show marked increases in these neurotransmitters following a single bout of exercise (Maddock et al., 2016). It is therefore conceivable that exercise into older age may address this excitatory-inhibitory imbalance and minimize impairments in force control.

It has been suggested that the age-related deterioration of the 1a-afferent reflex arc may be attenuated in Masters athletes (Unjehm et al., 2016). Germer et al. (2020) recently demonstrated that force steadiness significantly improved (i.e., CV decreased) in young adults when afferent feedback, in the form of sinusoidal vibrotactile stimulation, was introduced. This greater force steadiness was accompanied by a decrease in the variability of the smoothed cumulative MU spike train. These results suggest that, if lifelong physical activity maintains the integrity of afferent pathways, it may lead to a more stable oscillatory input to motor neurons and, consequently, the maintenance of force control.

Older adults exhibit lower (Connelly et al., 1999) and more variable (Tracy et al., 2005) MU discharge rates than young adults. It has recently been demonstrated that Masters athletes exhibit an increase in MU discharge rates following performance of a 21 km run, which was suggested to move MU firing properties closer to that exhibited by young adults (Cogliate et al., 2020). This acute effect of exercise has been extended to a more chronic timescale, with 6-week resistance training programs increasing MU discharge rates in both young (Vilã-Cha and Falla, 2016) and old adults (Kamen and Knight, 2004). Such physical activity-induced changes in discharge rate have been correlated with improvements in force steadiness (i.e., decreased CV) in both young (Vilã-Cha and Falla, 2016) and old adults (Kornatz et al., 2005). Furthermore, lifelong resistance training, but not lifelong recreational activity, has been demonstrated to attenuate an age-related decrease in efferent drive to muscle (Unjehm et al., 2016). It is possible, therefore, that the lifelong training characteristic of Masters athletes has a positive effect on neural drive to muscle, which may subsequently be reflected in the ability to control force.

### Peripheral Adaptations

The peripheral remodeling of MUs with advanced aging may be generally described as resulting in a decrease in number, and *via* compensatory expansion and “rescue” of denervated fibers, an increase in MU size (i.e., innervation ratio; Deschenes, 2011; Piasecki et al., 2016). Although no human data are available to confirm, rodent models have demonstrated that higher threshold (later recruited) MUs are more susceptible to loss with age, and lower threshold (earlier recruited) MUs have greater capacity to “rescue” denervated fibers (Kadhiresan et al., 1996). As MU size is also believed to correlate with achievable levels of fine control, i.e., those with fewer fibers generate finer movements (Azevedo et al., 2020), this increase in fiber ratio of the lower threshold MUs may be expected to impact upon force steadiness and may partly explain why the most prominent age-related differences are apparent at lower force levels (<10% MVC).

Masters athletes have greater remodeling capacity than non-athletic age-matched controls (Piasecki et al., 2019), theoretically creating a muscle environment where reinnervation of denervated fibers is more successful. This notion has been demonstrated *via* larger intramuscularly recorded MU potentials at normalized contraction intensities (Piasecki et al., 2019), more homogeneous potentials across muscle depths (Jones et al., 2021), fewer denervated fibers (Sonjak et al., 2019; Soendenbroe et al., 2021), and greater fiber type grouping (Zampieri et al., 2015) in Masters athletes compared to non-athletic age-matched controls. Although the latter is not a consistent finding and may be muscle and method dependent (Messa et al., 2020), the collective evidence support this concept of greater reinnervation. If a larger MU size, or a greater extent of remodeling of lower threshold MUs, is more apparent in Masters athletes it is conceivable that force control may be impaired in this group, perhaps even to a greater extent than in non-athletic age-matched controls. However, the magnitude of MU expansion is difficult to quantify and has a limited capacity in humans, and may, therefore, be insufficient to exert notable effects on force control.

Middle to older age (43–84 years) male and female Masters athletes showed a progressive age-related increase in markers of MU remodeling in the tibialis anterior, which was matched by a progressive increase in muscle force CV of the dorsiflexors at 10 and 25% MVC (Piasecki et al., 2021). These results demonstrate a clear association between MU remodeling and functional outputs, and highlight that extremely active people are not entirely spared from these neuromuscular decrements. However, there is still a lack of evidence as to whether the changes in muscle force control experienced by active and inactive adults with aging are quantitatively similar. Moreover, the magnitude-based measures of force control used in this study lack mechanistic insight and may reflect changes independent of MU remodeling and simply be inherent features of aging.

There is a lack of human data addressing the central and peripheral neural adaptations with aging and lifelong physical activity that contribute to muscle force control. This is in no doubt explained partly by a lack of appreciation of the effects physical activity has on muscle force control (by both researchers interested in force control and aging and in the effects of physical activity with aging) and partly by methodological limitations that are not apparent in animal models. Moreover, existing data may be further complicated by the oversimplification of MU types, sizes, and recruitment threshold, which in truth form a complex and overlapping trajectory rather than displaying distinct functional properties.

## Conclusion

It is apparent that lifelong physically active and inactive adults exhibit the same qualitative changes in muscle force control. However, given the accumulating evidence demonstrating differing physiology and functionality between these two distinct groups, future studies on the age-related loss of muscle force control must account for physical activity and make comparisons between active and inactive adults. This is particularly pertinent as there are plausible mechanisms by which lifelong physical activity may attenuate or worsen the age-related decrement in muscle force control.

## Data Availability Statement

The original contributions presented in the study are included in the article/supplementary material, further inquiries can be directed to the corresponding author.

## Author Contributions

All authors listed have made a substantial, direct, and intellectual contribution to the work and approved it for publication.

## Conflict of Interest

The authors declare that the research was conducted in the absence of any commercial or financial relationships that could be construed as a potential conflict of interest.

## Publisher's Note

All claims expressed in this article are solely those of the authors and do not necessarily represent those of their affiliated organizations, or those of the publisher, the editors and the reviewers. Any product that may be evaluated in this article, or claim that may be made by its manufacturer, is not guaranteed or endorsed by the publisher.
